# Risk factors for combined vaginal–cesarean delivery in the vertex–vertex twins: a case–control study

**DOI:** 10.1007/s00404-026-08406-2

**Published:** 2026-03-26

**Authors:** Itay Manor, Daniel Gabbai, Yoav Baruch, Yariv Yogev, Liran Hiersch, Emmanuel Attali

**Affiliations:** 1 Lis Hospital for Women, Tel Aviv Sourasky Medical Center, Weitzman 6 St., 6423906 Tel Aviv, Israel; 2 Gray Faculty of Medical & Health Sciences, Tel aviv University, Tel Aviv-Yafo, Israel

**Keywords:** Gestational diabetes (GDM), Birthweight discordance, Combined delivery, Twins

## Abstract

**Purpose:**

Twin pregnancies pose unique challenges during labor, particularly the risk of cesarean section for the second twin after vaginal delivery of the first—termed a *combined vaginal–cesarean delivery*. Although nonvertex presentation of the second twin is a recognized risk factor, predictors in vertex–vertex twin pregnancies remain unclear. The objective of this study was to identify maternal, pregnancy-related, intrapartum, and fetal risk factors associated with combined vaginal–cesarean delivery in twin pregnancies with vertex–vertex presentation.

**Methods:**

A retrospective single-center case–control study was conducted at Lis Maternity and Women’s Hospital, Tel Aviv Sourasky Medical Center, between 2012 and 2023. The study included women with twin pregnancies in vertex–vertex presentation who attempted vaginal delivery. The case group comprised women who underwent combined vaginal–cesarean delivery (vaginal birth of the first twin followed by cesarean of the second), while controls delivered both twins vaginally. Maternal, pregnancy-related, intrapartum, and fetal variables were analyzed using univariate and multivariable logistic regression models.

**Results:**

Among 503 twin deliveries meeting inclusion criteria, 22 (4.4%) resulted in combined vaginal–cesarean delivery. Maternal characteristics, labor features, and fetal parameters were similar between groups. Gestational diabetes mellitus was significantly more frequent among combined deliveries (31.8% vs. 10.8%; *p* = 0.009) and remained the only independent risk factor after adjustment (odds ratio 4.06; 95% CI 1.57–10.53; *p* = 0.004). No other associations were found between the delivery outcome and other factors, including maternal age, parity, previous cesarean delivery, preeclampsia, instrumental delivery of the first twin, gestational age at delivery, and intertwin birthweight discordance.

**Conclusions:**

In vertex–vertex twin pregnancies, successful vaginal delivery is achieved in approximately 95% of cases. Gestational diabetes mellitus independently increases the likelihood of cesarean delivery for the second twin, suggesting a possible metabolic influence on labor dynamics. Optimal glycemic control and vigilant intrapartum management may help minimize cesarean conversions. Larger multicenter studies are warranted to validate these findings and explore the effect of diabetes severity and treatment type.

## What does this study add to the clinical work?

**Table:** 

Vaginal delivery is successful in approximately 95% of vertex–vertex twin pregnancies, supporting a trial of labor when conditions are favorable. For the first time, we demonstrate that gestational diabetes mellitus is an independent risk factor for cesarean delivery of the second twin, highlighting a potential metabolic influence on labor and the importance of optimized glycemic control and intrapartum vigilance.	

## Introduction

The rate of twin gestations has risen over recent decades, now accounting for approximately 3% of all pregnancies [1, 2]. This increase is primarily attributed to higher maternal age and the greater use of assisted reproductive technologies, despite advances in fertility care and updated professional guidelines aimed at reducing multiple pregnancies [3].

Twin pregnancies present unique challenges during labor and delivery. Cesarean section rates for twin deliveries have increased significantly over time, reaching up to 75% in some populations, with breech presentation and previous cesarean delivery being the main contributors [4–6]. This trend can be partly explained by the influence of the Term Breech Trial [7], which, although focused on singleton pregnancies, led to a more conservative approach to vaginal delivery in twins. Nevertheless, the Twin Birth Study [8] demonstrated that planned vaginal delivery is both appropriate and safe when the first twin presents in vertex position, particularly when both twins are vertex [9, 10].

A significant concern among women and clinicians considering vaginal delivery of twins is the potential need for a cesarean section for the second twin following vaginal delivery of the first, a scenario known as a combined vaginal–cesarean delivery. This outcome is reported in approximately 4–10% of cases [8, 11–17], and specific prediction tools have even been developed to estimate this risk [6].

Although nonvertex presentation of the second twin is an established risk factor for combined delivery, uncertainty remains regarding additional predictors, particularly when both twins present as vertex [6, 12–19].

Previous studies have suggested that several maternal, pregnancy-related, intrapartum, and fetal factors may influence the likelihood of a combined vaginal–cesarean delivery in twin pregnancies, including a nonvertex second twin. For example, factors such as a history of cesarean section, instrumental delivery of the first twin, preterm birth, and birthweight discordance have been proposed as potential contributors in some studies [6, 13–15], whereas others have found no significant association [11, 12, 14, 17–19]. This inconsistency highlights the need for a better understanding of the factors that may increase the risk of conversion to cesarean section for the second twin. Clarifying these risk factors could improve patient counseling, optimize labor management, and help reduce unnecessary cesarean deliveries.

The present study seeks to address this clinical gap by identifying risk factors for combined vaginal–cesarean delivery in women with twin pregnancies with vertex–vertex presentation who had a vaginal delivery of the first twin.

## Methods

### Study design and population

This retrospective case–control study was conducted at Lis Maternity & Women’s Hospital, Tel Aviv Sourasky Medical Center, between January 1, 2012, and December 31, 2023.

The study was approved by the Helsinki Committee of Tel Aviv Sourasky Medical Center (approval number TLV-0284–08).

The study included only women with twin pregnancies in vertex–vertex presentation who underwent a trial of vaginal delivery during the study period.

The case group consisted of women who underwent a combined vaginal–cesarean delivery, defined as vaginal birth of the first twin followed by cesarean delivery of the second twin.

The control group included women who successfully delivered both twins vaginally.

Women were excluded if they had a cesarean delivery for both twins, a nonvertex presentation of one or both fetuses at the onset of labor or missing or incomplete data regarding delivery mode or fetal presentation.

**Fig. 1:**
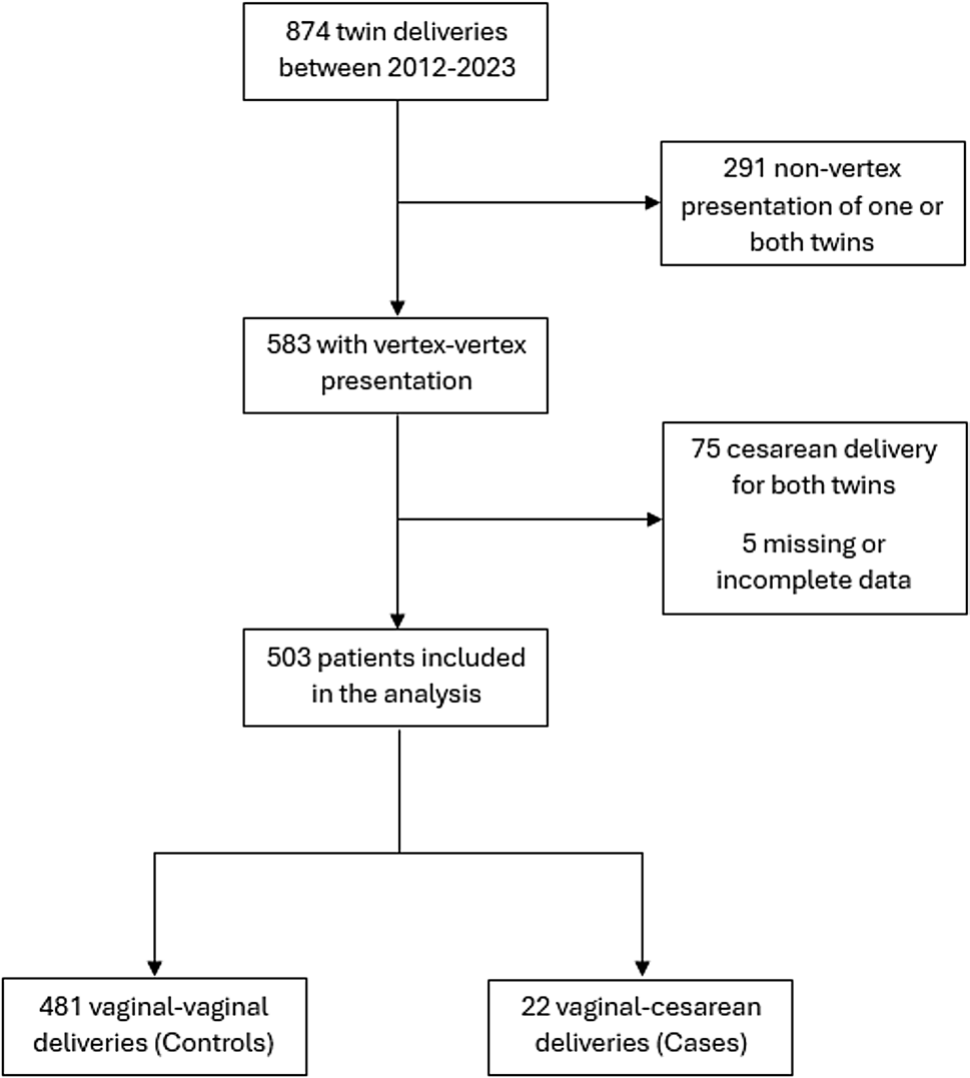
Flow diagram of study population selection

During the study period, 874 twin deliveries were identified. After exclusion of cases with nonvertex presentation of one or both fetuses (*n* = 291), cesarean delivery for both twins (*n* = 75), and missing or incomplete data (*n* = 5), 503 vertex–vertex twin deliveries remained for analysis. The selection process is illustrated in Fig. 1.

### Data collection and variables

Data was obtained from the hospital’s computerized data system, which compiles standardized electronic medical records that are continuously updated by medical and nursing staff throughout hospitalization to ensure accuracy and completeness.

Data collection was guided by previously published literature and by the availability of reliable data within the institutional database. Collected variables included maternal, pregnancy-related, intrapartum, and fetal characteristics:

- **Maternal factors:** age, prepregnancy body mass index (BMI), gestational weight gain, nulliparity, previous cesarean section, recurrent pregnancy loss, and the presence of chronic diabetes.
- **Pregnancy-related factors:** in vitro fertilization (IVF), gestational diabetes mellitus (GDM), diagnosed using a 50-g glucose challenge test followed by a diagnostic 100-g oral glucose tolerance test, and preeclampsia.
- **Intrapartum factors:** mode of labor onset (spontaneous, induction, or augmentation), use of epidural anesthesia, intrapartum fever, and instrumental delivery of the first twin
- **Fetal factors:** gestational age at delivery and birthweight discordance between the twins.

The primary outcome was the occurrence of a combined vaginal–cesarean delivery.

### Statistical analysis

All variables were classified by measurement scale, and their distributions were examined. Continuous variables were evaluated for normality using histograms, Q–Q plots, and the Kolmogorov–Smirnov test.

A univariate analysis was conducted to compare the case and control groups. Continuous variables were compared using the independent-samples *t*-test when normally distributed; otherwise, non-parametric tests were used. Ordinal and categorical variables were presented as frequencies and compared using parametric or chi-square tests, depending on the variable type.

Variables that were statistically significant in univariate analysis were entered into the multivariable model. Maternal age and gestational age at delivery were also included a priori because of their recognized clinical relevance and potential confounding effects.

All tests were two-tailed and based on a 95% confidence interval (CI = 95%), with *p* < 0.05 considered statistically significant.

## Results

A total of 503 women met the study’s inclusion criteria between January 2012 and December 2023. All had twin pregnancies in vertex–vertex presentation and a vaginal delivery of the first twin. Among them, 22 (4.4%) underwent a combined vaginal–cesarean delivery, while 481 (95.6%) delivered both twins vaginally. The results are summarized in Table 1.

**Table 1:** Characteristics of vertex–vertex twin deliveries by mode of delivery

Variable	Cases (*n* = 22)	Controls (*n* = 481)	*p* value
Maternal characteristics			
Maternal age, mean ± SD (y)	32.1 ± 5.4	33.0 ± 4.6	0.231
BMI at follow-up, median [IQR] (kg/m^2^)	22.1 [19.7–24.9]	22.1 [20.2–24.7]	0.736
Gestational weight gain, median [IQR] (kg)	13 [10–17]	14 [10–17]	0.697
Nulliparity, *n* (%)	11 (50.0)	192 (39.9)	0.379
Previous cesarean section, *n* (%)	0 (0)	6 (1.2)	1.000
Recurrent pregnancy loss, *n* (%)	2 (9.1)	18 (3.7)	0.216
Pregnancy-related factors			
In-vitro fertilization, *n* (%)	9 (40.9)	137 (29.7)	0.341
Gestational diabetes mellitus, *n* (%)	7 (31.8)	52 (10.8)	0.009
Preeclampsia, *n* (%)	1 (4.5)	15 (3.1)	0.517
Chronic diabetes, *n* (%)	0 (0)	1 (0.2)	1.000
Intrapartum variables			
Onset of labor, *n* (%)			0.352
Spontaneous	13 (59.1)	328 (67.8)	
Induction	9 (40.9)	138 (28.7)	
Augmentation	0 (0)	17 (3.5)	
Epidural anesthesia, *n* (%)	18 (81.8)	427 (89.0)	0.298
Intrapartum fever, *n* (%)	2 (14.3)	20 (5.9)	0.212
Instrumental delivery of first twin, *n* (%)	2 (9.1)	37 (7.7)	0.685
Fetal parameters			
Gestational age at delivery, median [IQR] (wk)	37.8 [36.1–38.3]	37.2 [35.6–38.1]	0.125
Intertwin birthweight difference, median [IQR] (g)	198 [119–398]	197 [80–352]	0.351

Maternal characteristics were broadly similar between groups. The mean maternal age was 32.1 ± 5.4 years among women who had a combined vaginal–cesarean delivery and 33.0 ± 4.6 years among those with a successful vaginal birth of both twins (*p* = 0.231). The median body mass index at follow-up was 22.1 kg/m^2^ (IQR 19.7–24.9) in cases and 22.1 kg/m^2^ (IQR 20.2–24.7) in controls (*p* = 0.736). Median gestational weight gain was 13 kg (IQR 10–17) versus 14 kg (IQR 10–17; *p* = 0.697). Nulliparity was somewhat more common in cases, yet still not statistically significant (11 of 22 [50.0%] vs 192 of 481 [39.9%]; *p* = 0.379). Previous cesarean section was recorded in none of the cases and in 6 women from the control group (*p* = 1.000), while recurrent pregnancy loss was documented in 2 (9.1%) and 18 (3.7%), respectively (*p* = 0.216). Chronic diabetes was rare, occurring in only one woman in the control group and in none of the cases (*p* = 1.000).

Pregnancy-related characteristics revealed only one statistically significant difference between the two groups. Rates of in vitro fertilization were comparable (9 of 22 [40.9%] vs. 137 of 481 [29.7%]; *p* = 0.341), as were rates of preeclampsia (1 [4.5%] vs. 15 [3.1%]; *p* = 0.517). In contrast, gestational diabetes mellitus (GDM) was markedly more prevalent among women who required cesarean delivery for the second twin, occurring in 7 of 22 (31.8%) compared with 52 of 481 (10.8%) in the control group (*p* = 0.009).

Intrapartum variables did not differ substantially between groups. Spontaneous onset of labor was observed in 13 women (59.1%) with combined delivery and 328 (67.8%) in the control group, while induction occurred in 9 (40.9%) and 138 (28.7%) deliveries, respectively; no augmentations were recorded among cases, compared with 17 (3.5%) in controls. The overall distribution of labor onset did not differ significantly between groups (*p* = 0.352). Epidural anesthesia was administered in 18 (81.8%) and 427 (89.0%) women (*p* = 0.298). Intrapartum fever occurred in 2 (14.3%) and 20 (5.9%), and instrumental delivery of the first twin was infrequent—2 (9.1%) versus 37 (7.7%) (*p* = 0.685).

Fetal parameters were similar across groups. The median gestational age at delivery was 37.8 weeks (IQR 36.1–38.3) among cases and 37.2 weeks (IQR 35.6–38.1) among controls (*p* = 0.125). The median intertwin birthweight difference was almost identical—198 g (IQR 119–398) among cases and 197 g (IQR 80–352) among controls (*p* = 0.351).

A multivariable logistic regression was then performed to identify independent predictors of combined vaginal–cesarean delivery (Table 2). GDM was included as the only variable showing statistical significance in univariate analysis. Maternal age and gestational age at delivery were added a priori as described in the Methods. After adjustment, GDM remained the only independent risk factor for combined vaginal–cesarean delivery (odds ratio [OR] 4.06, 95% CI 1.57–10.53; *p* = 0.004).

**Table 2:** Multivariable analysis of risk factors for cesarean delivery of the second twin

Variable	Odds ratio (95% CI)	*p* value
Maternal age (per year)	0.95 (0.87–1.04)	0.307
Gestational age at delivery (per week)	1.16 (0.91–1.47)	0.231
Gestational diabetes mellitus	4.06 (1.57–10.53)	0.004

In summary, among 503 twin deliveries with vertex–vertex presentation, the overall rate of combined vaginal–cesarean delivery was 4.4%. Across all evaluated maternal, pregnancy, intrapartum, and fetal parameters, gestational diabetes mellitus emerged as the sole independent predictor of cesarean delivery for the second twin.

## Discussion

In this retrospective case–control study of 503 vertex–vertex twin deliveries, we found a combined vaginal–cesarean delivery rate of 4.4%, consistent with the lower end of previously reported rates [11, 12, 14, 15, 17–19]. Gestational diabetes mellitus (GDM) was the only independent risk factor, conferring about a fourfold increase in odds. No other maternal, pregnancy, intrapartum, or fetal factors were significantly associated.

Because nonvertex presentation of the second twin is the most substantial known risk factor for combined delivery [6, 12–15, 17–19], we focused exclusively on vertex–vertex pregnancies to remove this confounder. Restricting analysis to these cases enabled assessment of subtler factors, as evidence for them remains inconclusive.

Maternal characteristics showed inconsistent associations with combined delivery. Previous studies reported conflicting findings regarding parity and prior cesarean delivery, with some demonstrating higher rates among women with a uterine scar [15] or multiparity [18], whereas others found no association [12, 17, 19]. Similarly, although advanced maternal age has been linked to higher overall intrapartum cesarean rates in twin pregnancies [6], it has not been consistently associated with cesarean delivery of the second twin [13, 18]. In our cohort, neither parity, prior cesarean delivery, nor maternal age was significantly associated with combined delivery.

Among pregnancy factors, GDM was the only significant predictor. It occurred in nearly one-third of women who required cesarean for Twin B, compared with about 11% among those with complete vaginal deliveries. To our knowledge, this is the first study to identify GDM as an independent risk factor for combined delivery in vertex–vertex twins. Both Wen et al. [14] and Yang et al. [11] found that maternal “medical complications,” including GDM, increased the risk for second-twin cesarean. Still, they did not analyze the effect of each complication separately. In contrast, Spiegel et al. [15] also examined maternal comorbidities and found no association between GDM and combined delivery. Our findings thus expand existing knowledge, suggesting that maternal metabolic status may influence twin labor outcomes.

Other pregnancy-related factors were not associated with combined delivery. Hypertensive disorders showed no relationship in our cohort, in contrast to isolated findings in a previous study based on a small sample [15]. Similarly, although infertility treatment has been associated with cesarean delivery of the second twin in some reports [13], we observed only a non-significant trend.

No intrapartum factors were significantly associated with combined delivery. Previous studies have reported inconsistent findings regarding labor onset and operative delivery of the first twin [6, 13, 14]. In our cohort, neither labor onset nor instrumental delivery of the first twin influenced the likelihood of cesarean delivery for the second twin.

A previous study [18] found the spontaneous version of Twin B to be more common among women requiring cesarean in univariable analysis, although this association was not significant after adjustment. Some of our combined cases may therefore reflect unrecorded malpresentation or cord prolapse, both recognized causes of emergent cesarean delivery [11, 14]. However, information on these indications was not available in our dataset.

Among fetal factors, gestational age at delivery was not associated with combined delivery in our cohort. Previous studies have reported conflicting results: some linked preterm delivery with higher risk [6, 14, 15], whereas others [13] reported greater risk beyond 39 weeks. Others [11, 12, 17–19] found no association.

Birthweights and intertwin birthweight discordance were comparable between groups, suggesting that the increased risk associated with GDM was not mediated through fetal size differences. Previous studies reported that when the second twin was ≥ 25% heavier than the first, the risk of combined vaginal–cesarean delivery increased significantly [11, 14], whereas others found no such association [12, 13, 17, 19]. Extreme discordance was not observed in our cohort, suggesting that moderate discordance alone does not increase the risk. It should be noted that our analysis relied on birthweights, whereas clinical management typically depends on ultrasound-estimated fetal weights, which were unavailable in our dataset.

Several mechanisms may explain the link between GDM and combined delivery. Although fetal size did not differ, polyhydramnios and metabolic or vascular changes associated with GDM may predispose to malpresentation, cord prolapse, or fetal distress, prompting cesarean. Clinicians may also intervene earlier due to concerns about complications. Together, these factors could explain the higher combined-delivery rate observed among women with GDM.

This study has several strengths. It is one of the few analyses limited to vertex–vertex twin gestations, eliminating a major confounder and allowing more precise assessment of other factors. Data from 2012 to 2023 provided internal consistency and reflected modern practice. The combined-delivery rate matched other contemporary reports, supporting generalizability. Most importantly, identifying GDM as a novel, independent risk factor provides new insights into the pathophysiology of labor in twin pregnancies and may inform future research and clinical counseling.

Several limitations should be noted. The relatively small number of cases (*n* = 22) reduces statistical power to detect moderate associations and increases the risk of type II error. In addition, it may affect the stability of multivariable logistic regression models and raise the possibility of model overfitting; therefore, the results should be interpreted cautiously. Some important variables were unavailable or inconsistently documented in the dataset. These included the specific clinical indications for cesarean delivery of the second twin, as well as detailed information regarding GDM severity, treatment modality (diet versus insulin), and degree of glycemic control, limiting further analysis of the mechanisms underlying the observed association. Selection bias is another consideration: women with advanced age, comorbidities, significant discordance, or prior cesarean delivery might have been scheduled for elective cesarean delivery and thus excluded, leaving a lower-risk group. This selection may have reduced the overall incidence of combined delivery and limited the ability to detect associations with some maternal or obstetric risk factors. Finally, as a single-center study, our results reflect tertiary-level management with immediate surgical availability, which may limit external validity.

Although not a limitation per se, it is worth noting that neonatal outcomes were not analyzed, as they were beyond the scope of this study. Nevertheless, these outcomes are often central to patient counseling and clinical decision-making and should be addressed in future research.

In conclusion, our findings confirm that in vertex–vertex twin pregnancies, successful vaginal delivery is common (~ 95%), supporting a trial of labor when conditions are favorable. While most maternal, fetal, and intrapartum variables were not significantly associated with combined vaginal–cesarean delivery, gestational diabetes mellitus emerged as an independent risk factor, suggesting a potential metabolic influence on delivery dynamics. This highlights the importance of optimal glycemic control and vigilant intrapartum monitoring. For women with twins and GDM, counseling should acknowledge a slightly higher likelihood of cesarean for Twin B but emphasize the overall low absolute risk. Larger multicenter studies should validate these findings and examine whether glycemic control, treatment modality, or other metabolic factors modify this risk. Recognition and management of modifiable factors, such as GDM, may help reduce combined deliveries and improve maternal and neonatal outcomes.

## Data Availability

No datasets were generated or analyzed during the current study.
